# The Konno–Rastan procedure in children and young adults: efficiency versus effectiveness

**DOI:** 10.1093/icvts/ivad171

**Published:** 2023-10-17

**Authors:** Bahaaldin Alsoufi

**Affiliations:** Department of Cardiovascular and Thoracic Surgery, University of Louisville, Norton Children’s Hospital, Louisville, KY, USA

**Keywords:** Konno–Rastan procedure, Prosthetic valve, Aortic stenosis, Aortic regurgitation, Patient–prosthesis mismatch

In the current issue of the *Interdisciplinary Cardiovascular and Thoracic Surgery* (*ICVTS*) journal, Amirghofran *et al.* from Iran present their impressive series of 48 patients who underwent aortic valve replacement (AVR) with annular enlargement using the Konno–Rastan technique. The age range of these patients was 2–53 years. The mean preoperative diameter of the aortic valve annulus was 15 mm, and the mean enlargement of the annulus with Konno–Rastan was 5 mm. Since the mean valve annulus was 15 mm, the authors divided their cohort into 2 groups: those <15 and >15 mm prior to operation (*n* = 19 and 29, respectively). Overall, they demonstrated an immediate reduction of gradient from 79 to 21 mmHg. They reported low operative mortality (2%), low incidence of heart block (4%) and fast recovery with a mean hospital stay of 8 days. Interestingly, all the complications were in the group with aortic annulus <15 mm, with operative mortality and heart block risk of 5% and 11%, respectively. While they reported no valve-related reoperations, no bleeding or thromboembolic complications and what seems to be stable gradients, there were 2 late deaths, both in the group with aortic anulus <15 mmHg [1].

Undeniably, the results reported by the group from Iran are impressive. Despite the complexity of these patients, with many of them having prior or simultaneous cardiac reoperations, early outcomes and recovery times were remarkable. This might be attributed to patient selection; however, there is no doubt that the authors have performed very efficient and well-organized surgeries with excellent anatomic and haemodynamic results. Despite the frequent association with concomitant cardiac operations, the mean cardiopulmonary bypass and aortic cross-clamp times were impressively low at 102 and 86 min, respectively. The accompanying video is a clear demonstration of the authors’ capabilities that definitely contributed to their good early outcomes [1].

The authors from Iran have demonstrated that AVR with Konno–Rastan enlargement can be done in many patients, including children as young as 2 years of age. The question arises, however, is that the best option for everyone? Most importantly, is that the best option for small children? Efficiency is doing things right; however, effectiveness is doing the right thing. As stated above, the authors were clearly efficient performing this complex operation with good early outcomes. I would challenge, however, if AVR with Konno–Rastan is the optimal operation for small children. I would argue that, while not perfect, AVR using the Ross–Konno operation might still be a superior choice in small children who have a functional pulmonary valve. Operative mortality following Ross or Ross–Konno operation is extremely low in children >1 year of age and might be better than that reported in the younger cohort in the series from Iran [2–5]. Additionally, haemodynamics following Ross–Konno are naturally superior and the expected residual gradients usually approach zero and in general remain that low. While the immediate gradient following Konno–Rastan in the series from Iran was about 21 mmHg, this is unlikely to remain that low with follow-up as the child grows. The authors report that the mean gradient on the last follow-up was acceptable in most patients; however, they do not discriminate between smaller and older patients, and they do not provide the duration of follow-up of the last echocardiograms. A valve size that is 19 mm in diameter might prove to be adequate for the first few years but will likely be associated with the development of patient–prosthesis mismatch and related increased gradients and subsequent ventricular hypertrophy that would compromise the long-term benefit of AVR in these children. While late mortality seems to be low in this series, it was mainly in the aortic annulus <15 mm group, understanding that one of these deaths was likely not cardiac-related. This is in contrast to the Ross procedure where late attrition seems to be minimal [2–5]. Similarly, while not very excessive, the risk of heart block seems to be lower in reported series of Ross–Konno than that reported in the smaller patient cohort in the series from Iran (11%). Ross–Konno modifications with limited enlargement of the annulus without the use of ventricular septal defect patch might contribute to the lower risk of heart block [3]. The authors reported no valve-related cardiac reoperations within the follow-up duration; however, I remain sceptic if that will remain true in the smaller patient population with longer follow-up. Reoperation following Konno–Rastan is naturally challenging and might be associated with high mortality and morbidity, including heart block and bleeding [6]. While Ross–Konno in small children is associated with reoperation for conduit replacement, autograft reoperation seems to be lower in the younger population for unclear reasons [2–5]. One theory is related to the fact that these patients commonly receive the Ross–Konno operation for aortic stenosis that is potentially associated with a lower risk of future autograft dilatation and insufficiency. Another theory is related to the earlier use of the autograft that might be more adapt, due to elevated pulmonary vascular pressures, to be used in the systemic location; hence, the higher freedom from autograft reoperation. Additionally, the modified Ross–Konno, without the use of ventricular septal defect patch, might preserve the support system underneath the autograft and decrease the risk of future autograft dilatation [3]. One final point is related to anticoagulation challenges in children. While the authors report no anticoagulation-related complications during their follow-up, we know that this is not the case in reports from developed and developing countries alike. The Ross–Konno does not require anticoagulation and that might provide patients with a better lifestyle, and decreased potential complication risk [2–5].

In summary, there is a difference between efficiency and effectiveness. Being able to do the procedure proficiently does not necessarily mean that this was the most appropriate procedure to be done. While acknowledging that there is no perfect AVR choice, the role of the surgeon is to offer the procedure that is not only associated with good early outcomes but also with the best cardiac recovery, late survival and quality of life. I feel that Ross–Konno might continue to be the better AVR option in smaller children who are unable to receive an adult-size prosthesis at the time of their initial operation.
